# Etiological agents of bacterial sepsis in a newly constructed medical center in Saint Petersburg, Russia

**DOI:** 10.1186/cc10414

**Published:** 2011-10-27

**Authors:** E Barantsevich, N Barantsevich, N Rybkova, I Churkina, N Pestova, M Karpenko

**Affiliations:** 1 Almazov Federal Center of Heart, Blood and Endocrinology, Saint Petersburg, Russia

## Introduction

Sepsis is one of the factors of high mortality in ICUs in critically ill patients. Annual mortality from this condition is estimated at 30 to 50 deaths per 100,000 population [1,2,4,5]. The aim of the present study was to reveal the spectrum and resistance to antibiotics of microorganisms, causing sepsis in hospitalized patients of the multidisciplinary medical center, that accumulates patients from all regions of Russia, during the first year from its foundation, with low possibility of local nosocomial strains formation.

## Methods

The diagnosis of sepsis was verified by isolation of bacteria from blood (only two or more positive results were considered) and the presence of two or more criteria of systemic inflammatory response syndrome [3]. The cultures were isolated from blood with BactAlert (BioMerieux, France). The identification was performed by routine phenotypic methods and sequencing of the 16sRNA gene (ABI Prism 3130, MicroSeq ID v2.0 Software, MicroSeq ID 16s rDNA500 Library v2.0). Resistance to routinely used antibiotics was investigated with the disc diffusion method and by dilution techniques for MIC determination on Muller-Hinton agar (Oxoid, UK).

## Results

Sepsis, associated with bloodstream infections, was revealed in 89 cases - Gram-positive cocci predominated. *Staphylococcus *spp. were responsible for 35 (39.3%) cases: *Staphylococcus aureus *was the causative agent in eight (8.9%), coagulase-negative staphylococci (*Staphylococcus epidermidis*, *Staphylococcus haemolyticus*, *Staphylo-coccus hominis novobiosepticus*) in 27 (30.3%) infections. Other Gram-positive cocci were *Enterococcus faecalis *in eight (8.9%), *Enterococcus faecium *in six (6.7%). Gram-negative microorganisms included *Acineto-bacter baumannii *that was found in 11 (12.4%), *Klebsiella *spp. (*Klebsiella pneumonia*, *Klebsiella rhinoscleromatis*, *Klebsiella oxytoca*) in five (5.6%), *Escherichia coli *in eight (8.9%), *Enterobacter *spp. (*Enterobacter cloaceae*, *Enterobacter aerogenes*, *Enterobacter hormaechea*) in three (3.4%), *Pseudomonas aeruginosa *in two (2.2%) patients with sepsis. Rarely isolated bacteria were *Bacillus thuringiensis *in one (1.1%), *Stenotrophomonas maltophylia *in one (1.1%), *Pantoeae agglomerans *in one (1.1%), *Corynebacterium mucifaciensis *in one (1.1%) case. Two and more species were isolated from blood in seven (7.9%) patients. In total, 105 strains were isolated in sepsis cases. Resistance to antibiotics was observed in 96 (91.4%) bacterial isolates; 56 (53.3%) were multidrug-resistant strains. All *E. faecium*, and nine (81.8%) strains of *A. baumannii *were resistant to seven or more antibiotics. All *E. faecium *strains were susceptible to linezolide, *A. baumannii *to tigecycline. Methicillin resistance was detected in two (15.4%) strains of *S. aureus *and 18 (60.0%) strains of coagulase-negative staphylococci; four (66.7%) strains of *E. faecium *were vancomycin resistant.

## Conclusion

Gram-positive bacteria were the leading causative agents of sepsis, associated with bloodstream infections in the newly-constructed hospital in Saint Petersburg. The majority of strains (91.4%) were resistant to antibiotics, and more than half of the isolates were multidrug resistant. Methicillin resistance was observed predominantly in coagulase-negative staphylococci, vancomycin resistance in *E. faecium*. All polyresistant *E. faecium *strains were susceptible to linezolide, *A. baumannii *to tigecycline.
